# Supplementary material for: VEGF/ERK activation and PI3K inhibition together drive a vein-to-artery transition in an in vitro model of human angiogenesis
Source: bioRxiv. 2025 Dec 19:2025.12.17.694993. Preprint. [Version 1] doi: 10.64898/2025.12.17.694993 (PMC12724401; doi:10.64898/2025.12.17.694993)
Supplement: 1 [file NIHPP2025.12.17.694993V1-supplement-1.pdf]

Figure S1

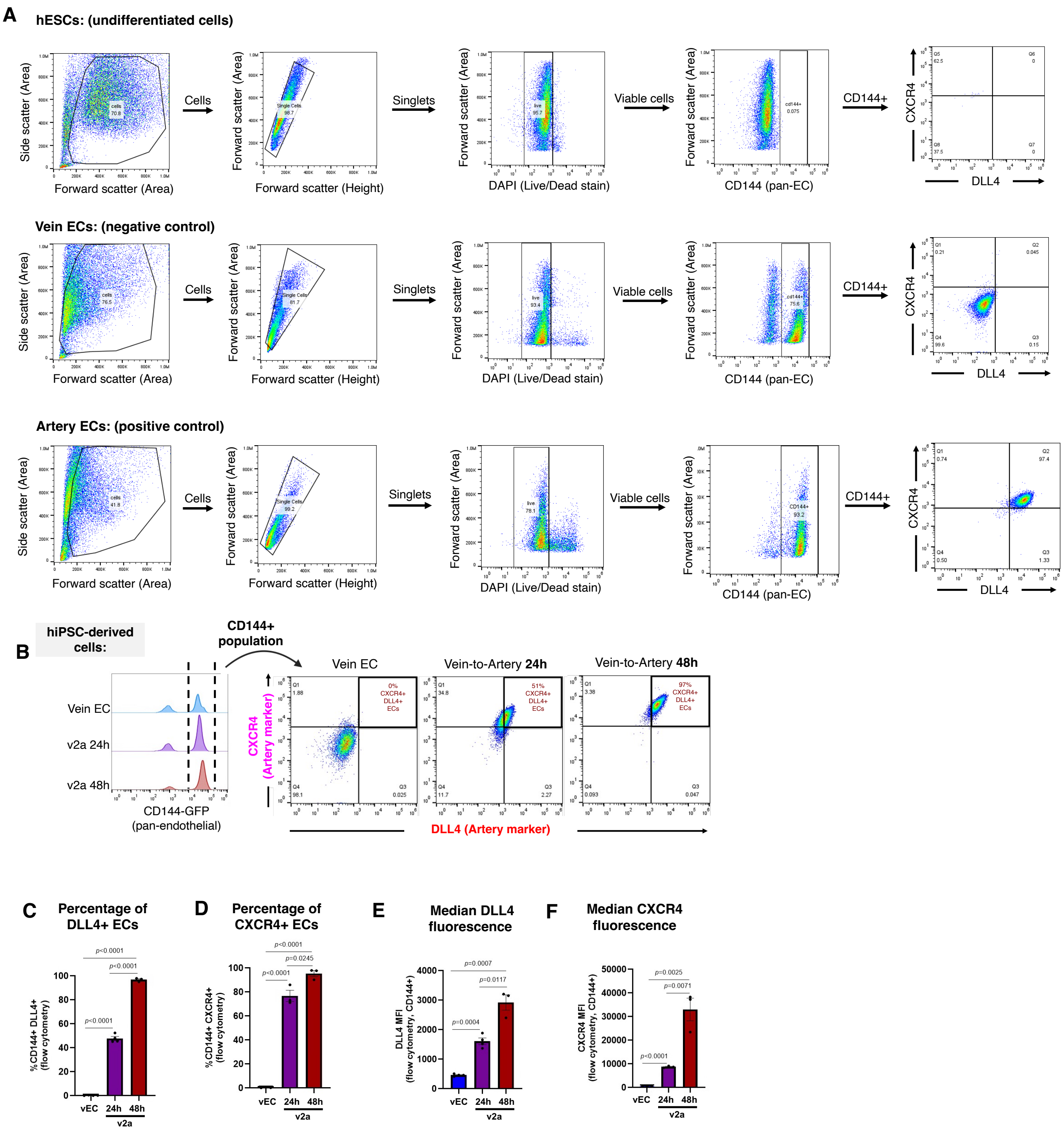
A
hESCs: (undifferentiated cells)
Vein ECs: (negative control)
Artery ECs: (positive control)
Side scatter (Area)
Forward scatter (Area)
Forward scatter (Height)
Cells
Singlets
Viable cells
CD144+
DAPI (Live/Dead stain)
CD144 (pan-EC)
CXCR4
DLL4
B
hiPSC-derived cells:
CD144+ population
Vein EC
v2a 24h
v2a 48h
CD144-GFP (pan-endothelial)
Vein EC
Vein-to-Artery 24h
Vein-to-Artery 48h
0% CXCR4+ DLL4+ ECs
51% CXCR4+ DLL4+ ECs
97% CXCR4+ DLL4+ ECs
CXCR4 (Artery marker)
DLL4 (Artery marker)
C
Percentage of DLL4+ ECs
% CD144+ DLL4+ (flow cytometry)
D
Percentage of CXCR4+ ECs
% CD144+ CXCR4+ (flow cytometry)
E
Median DLL4 fluorescence
DLL4 MFI (flow cytometry, CD144+)
F
Median CXCR4 fluorescence
CXCR4 MFI (flow cytometry, CD144+)
vEC
24h
48h
v2a

Figure S2

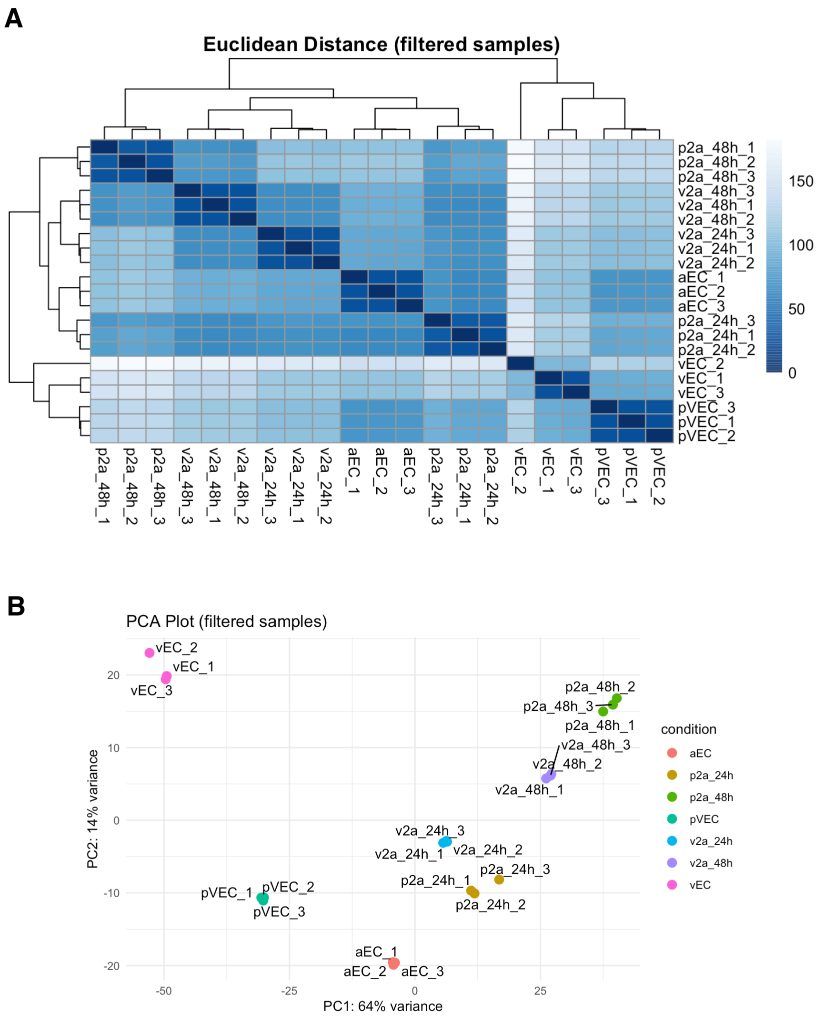

A
Euclidean Distance (filtered samples)
p2a_48h_1
p2a_48h_2
p2a_48h_3
v2a_48h_3
v2a_48h_1
v2a_48h_2
v2a_24h_3
v2a_24h_1
v2a_24h_2
aEC_1
aEC_2
aEC_3
p2a_24h_3
p2a_24h_1
p2a_24h_2
vEC_2
vEC_1
vEC_3
pVEC_3
pVEC_1
pVEC_2
150
100
50
0
B
PCA Plot (filtered samples)
PC2: 14% variance
PC1: 64% variance
condition
aEC
p2a_24h
p2a_48h
pVEC
v2a_24h
v2a_48h
vEC

Figure S3

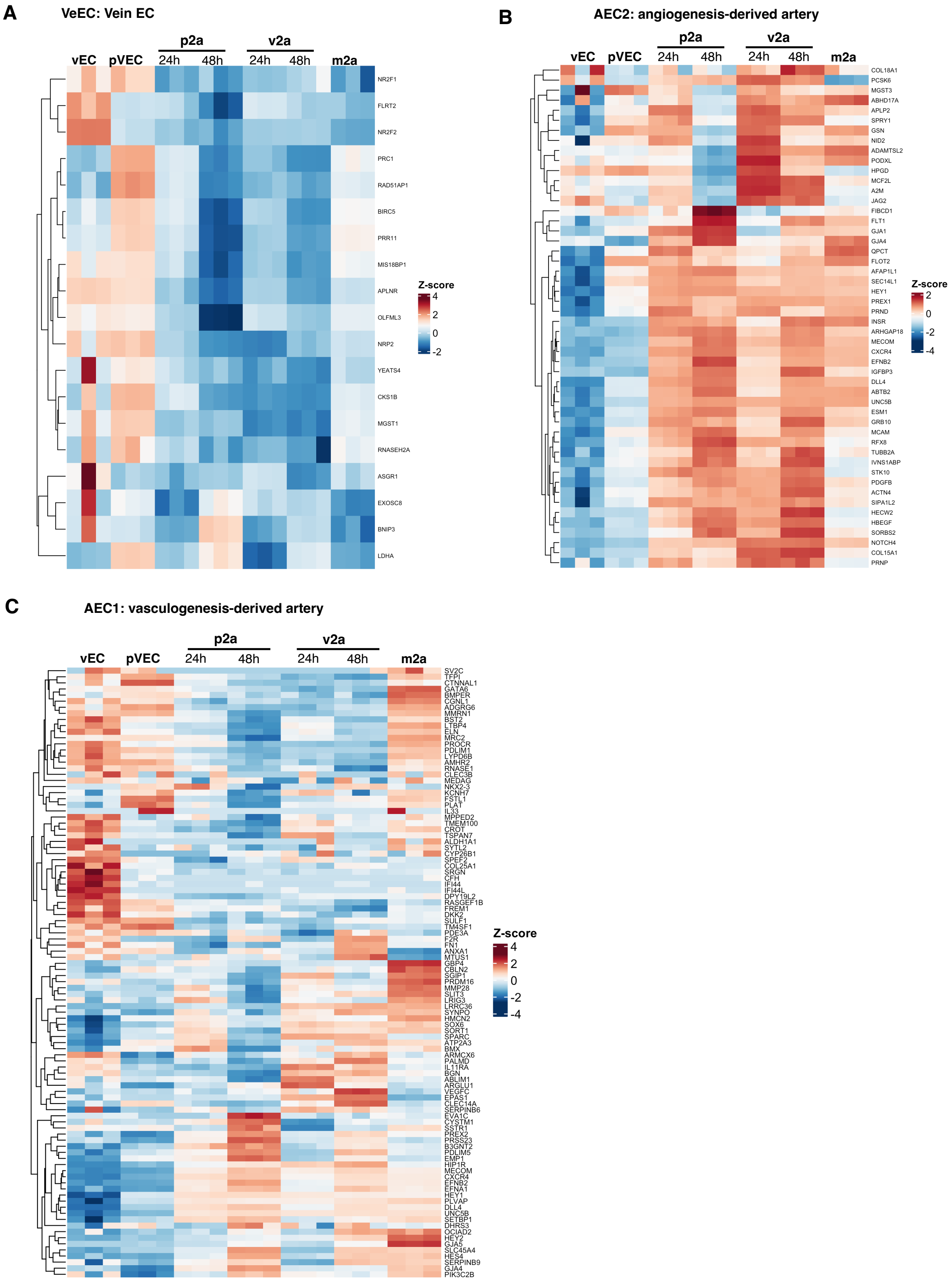
A
VeEC: Vein EC
vEC
pVEC
p2a
24h
48h
v2a
24h
48h
m2a
NR2F1
FLRT2
NR2F2
PRC1
RAD51AP1
BIRC5
PRR11
MIS18BP1
APLNR
OLFML3
NRP2
YEATS4
CKS1B
MGST1
RNASEH2A
ASGR1
EXOSC8
BNIP3
LDHA
Z-score
4
2
0
-2
B
AEC2: angiogenesis-derived artery
vEC
pVEC
p2a
24h
48h
v2a
24h
48h
m2a
COL18A1
PCSK6
MGST3
ABHD17A
APLP2
SPRY1
GSN
NID2
ADAMTSL2
PODXL
HPGD
MCF2L
A2M
JAG2
FIBCD1
FLT1
GJA1
GJA4
QPCT
FLOT2
AFAP1L1
SEC14L1
HEY1
PREX1
PRND
INSR
ARHGAP18
MECOM
CXCR4
EFNB2
IGFBP3
DLL4
ABTB2
UNC5B
ESM1
GRB10
MCAM
RFX8
TUBB2A
IVNS1ABP
STK10
PDGFB
ACTN4
SIPA1L2
HECW2
HBEGF
SORBS2
NOTCH4
COL15A1
PRNP
Z-score
2
0
-2
-4
C
AEC1: vasculogenesis-derived artery
vEC
pVEC
p2a
24h
48h
v2a
24h
48h
m2a
SV2C
TFPI
CTNNAL1
GATA6
BMPER
CGNL1
ADGRG6
MMRN1
BST2
LTBP4
ELN
MRC2
PROCR
PDLIM1
LYPD6B
AMHR2
RNASE1
CLEC3B
MEDAG
NKX2-3
KCNH7
FSTL1
PLAT
IL33
MPPED2
TMEM100
CROT
TSPAN7
ALDH1A1
SYTL2
CYP26B1
SPEF2
COL25A1
SRGN
CFH
IFI44
IFI44L
DPY19L2
RASGEF1B
FREM1
DKK2
SULF1
TM4SF1
PDE3A
F2R
FN1
ANXA1
MTUS1
GBP4
CBLN2
SGIP1
PRDM16
MMP28
SLIT3
LRIG3
LRRC36
SYNPO
HMCN2
SOX6
SORT1
SPARC
ATP2A3
BMX
ARMCX6
PALMD
IL11RA
BGN
ABLIM1
ARGLU1
VEGFC
EPAS1
CLEC14A
SERPINB6
EVA1C
CYSTM1
SSTR1
PREX2
PRSS23
B3GNT2
PDLIM5
EMP1
HIP1R
MECOM
CXCR4
EFNB2
EFNA1
HEY1
PLVAP
DLL4
UNC5B
SETBP1
DHRS3
OCIAD2
HEY2
GJA5
SLC45A4
HES4
SERPINB9
GJA4
PIK3C2B
Z-score
4
2
0
-2
-4

Figure S4

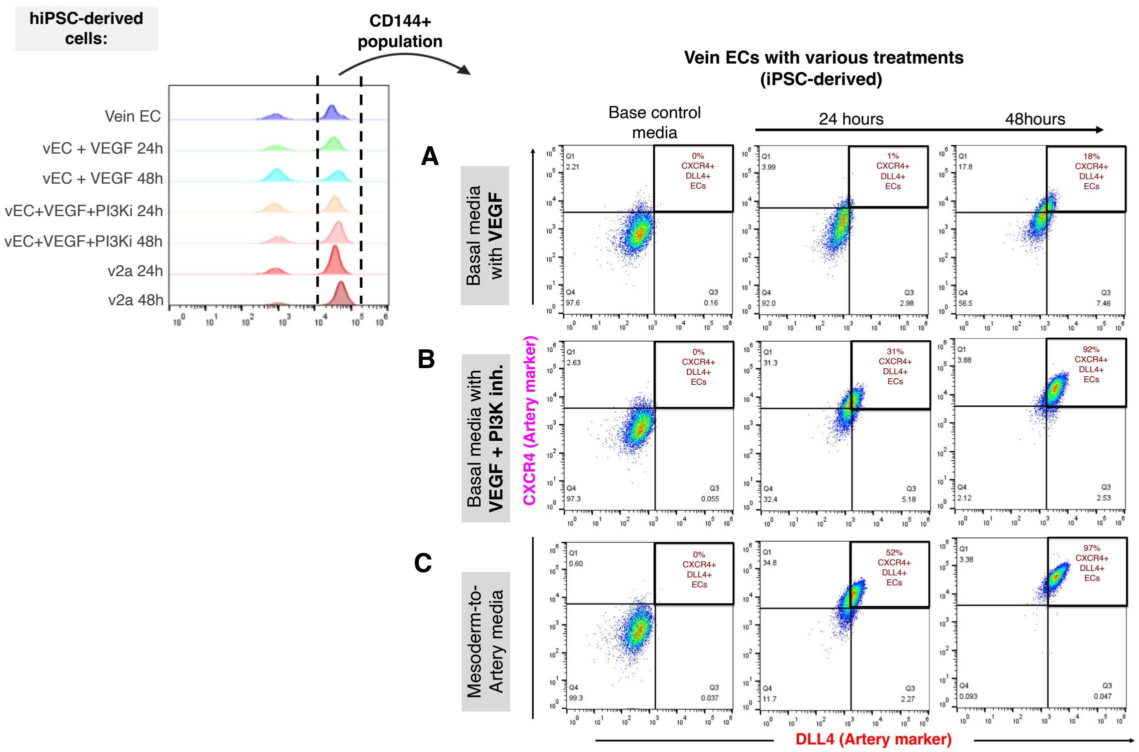
hiPSC-derived cells:
CD144+ population
Vein EC
vEC + VEGF 24h
vEC + VEGF 48h
vEC+VEGF+PI3Ki 24h
vEC+VEGF+PI3Ki 48h
v2a 24h
v2a 48h
Vein ECs with various treatments (iPSC-derived)
Base control media
24 hours
48hours
A
Basal media with VEGF
B
Basal media with VEGF + PI3K inh.
C
Mesoderm-to-Artery media
CXCR4 (Artery marker)
DLL4 (Artery marker)
0% CXCR4+ DLL4+ ECs
1% CXCR4+ DLL4+ ECs
18% CXCR4+ DLL4+ ECs
0% CXCR4+ DLL4+ ECs
31% CXCR4+ DLL4+ ECs
92% CXCR4+ DLL4+ ECs
0% CXCR4+ DLL4+ ECs
52% CXCR4+ DLL4+ ECs
97% CXCR4+ DLL4+ ECs

SUPPLEMENTAL FIGURE LEGENDS

Figure S1. Arterial induction of hPSC-derived venous endothelial cells.

(A) Representative gating strategy for undifferentiated human ESCs (negative for all markers). Vein ECs were used as a negative control for arterial markers (DLL4-APC, CXCR4-PE_Cy7) and as a positive control for pan-endothelial marker CD144-FITC.

(B) Representative flow cytometry plots depicting DLL4$^+$CXCR4$^+$ expression within CD144$^+$cells in hiPSC-derived vECs, and v2a ECs at 24 and 48 hours.

(C-F) Quantification of the percentage of CD144$^+$ ECs expressing DLL4$^+$ **(C)** and CXCR4$^+$ **(D)** populations in hiPSC-derived cells and their corresponding MFIs **(E, F)**, showing progressive arterial marker induction.

In histograms, each dot represents one independent experiment, and error bars are mean +/- SEM. *p*-values are shown.

Figure S2. Transcriptomic relationships among hPSC-derived endothelial cells and arterialized cells.

(A) Euclidean distance plot showing the global transcriptomic similarity across all samples, including arterial ECs (aEC), venous ECs (vEC), pre-venous ECs (pVEC), pre-vein-to-artery cells at 24 and 48 hours (p2a 24h and 48h), and vein-to-artery cells at 24 and 48 hours (v2a 24h and 48h).

(B) Principal component analysis (PCA) plot of the indicated samples, illustrating the variance and clustering of replicates for each cell type and time point. Each dot represents one technical replicate.

Figure S3. Human *in vivo* endothelial gene sets from Hou et al. applied to our *in vitro* samples.

(A-C) Heatmaps show the expression of the top genes from three human endothelial clusters defined by Hou et al.: VeEC (vein)**(A),** AEC2 (angiogenic arteries)**(B),** and AEC1 (vasculogenic arteries)**(C),** projected onto our in vitro samples (vEC, pVEC, p2a 24h and p2a 48h, v2a 24h and v2a 48h, and m2a ECs). These heatmaps show how venous ECs arterialize and acquire angiogenic and arterial identities during the vein-to-artery endothelial transition.

Figure S4. PI3K inhibition and VEGF are sufficient to arterialize vein endothelial cells in human iPSC-derived cells.

(A-C) Representative flow cytometry plots of CD144$^+$ DLL4$^+$CXCR4$^+$ cells across four treatment conditions.

REAGENT OR RESOURCE	SOURCE	IDENTIFIER
**Antibodies**		
APC anti-human Delta-like protein 4 (DLL4)	Biolegend	BioLegend Cat# 346508, RRID:AB_11204071
Mouse anti-human CD184 (CXCR4) monoclonal antibody, PE-Cy7 conjugated	BD Biosciences	BD Biosciences Cat# 560669, RRID:AB_1727435
Mouse anti-human CD144 monoclonal antibody, FITC conjugated	BD Biosciences	BD Biosciences Cat# 560411, RRID:AB_1645487
**Chemicals, peptides, and recombinant proteins**		
mTeSR Plus Medium	StemCell Technologies	100-0276
Penicillin/Streptomycin	Fisher Scientific	15-140-122
EGM2 MV Growth Medium Bullet Kit	Lonza	CC-3162
Bambanker Freezing Media	Fisher Scientific	NC2960954
Fetal Bovine Serum	Life/Thermo Fisher	A5670401
DAPI Staining Solution	Miltenyi Biotec	130-111-570
RNAlater Stabilizing Solution	Life/Thermo Fisher	AM7020
Geltrex LDEV-Free, hESC-Qualified Reduced Growth Factor Basement Membrane Matrix	Fisher Scientific	A1413302
Gelatin Solution	Sigma	ES-006-B
Versene Solution	Fisher Scientific	15040066
Accutase-Enzyme Cell Detachment Medium	Fisher Scientific	00-4555-56
TrypLE Express Enzyme (1X)	Fisher Scientific	12604013
2-mercaptoethanol	Gibco	21985023
DMEM	Sigma	D6429
F12 + GlutaMAX	Thermo Fisher	31765-092
IMDM + GlutaMAX	Thermo Fisher	31980-097

Polyvinyl Alcohol	Sigma	P8136-250G
Chemically Defined Lipid Concentrate	Thermo Fisher	11905-031
Recombinant Human Insulin	Sigma	11376497001
Recombinant Human VEGF	R&D Systems	293-VE-0500
1-thioglycerol	Sigma	M6145-100ML
Recombinant Human FGF2	R&D Systems	233-FB-01M
Human Transferrin	Sigma	10652202001
Recombinant Human BMP4	R&D Systems	314-BP-050
Recombinant Activin	R&D Systems	338-AC-500/CF
GDC-0941	Cellagen Technology	C4321-25
Forskolin	Tocris	1099
Thiazovivin	Tocris	3845
XAV939	Tocris	3748
Ascorbic Acid-2-Phosphate	Sigma	49752-10G
DMH1	Tocris	4126
SB505124	Tocris	3263
RO4929097	Cellagen Technology	C7649-10
CHIR99201	Tocris	4423
PD0325901	Tocris	4192
Bovine Serum Albumin (BSA)	Sigma	A2153
**Critical commercial assays**		
NucleoSpin RNA	Takara	740955.50
SYBRGreen	Thomas Scientific	BIO-94050
FcR Blocking Reagent	Miltenyi Biotec	130-059-901
Magnetically Activated Cell Sorting (MACS)	Miltenyi Biotec	130-046-703
CD34 MicroBead Kit, human	Miltenyi Biotec	130-046-702
MACS LS Column	Miltenyi Biotec	130-042-401
DPBS without ions	Thermo Fisher	14190144
Thermo Scientific Verso cDNA Synthesis Kit	Fisher/Life	AB1453B
**Experimental models: Cell lines**		
H1 human Embryonic Stem Cells (h1ESCs)	WiCell (gift from Loh Lab)	WiCell, WA01
Human Umbilical Vein Endothelial Cells (HUVEC)	Sigma-Aldrich	200-05N
Bi-Allelic mEGFP-tagged CDH5 WTC iPSC Line (TAG at C-Term) (hiPSC-Cdh5-GFP)	https://www.coriell.org	N/A

Oligonucleotides (Quantitative PCR Primers)

Primers	Forward	Reverse
DLL4	GTCTCCACGCCGGTATTGG	CAGGTGAAATTGAAGGGCAGT
SOX17	CGCACGGAATTTGAACAGTA	GGATCAGGGACCTGTCACAC
APLNR	CTCTGGACCGTGTTTCGGAG	GGTACGTGTAGGTAGCCCACA
NR2F2	GCCATAGTCCTGTTCACCTCA	AATCTCGTCGGCTGGTTG

CD144/VE-CADHERIN	AACGAGCAGGGCGAGTTCACCTTC	TAGGTGACCAGCTGCTCGTGGATC
YWHAZ	GAGCTGGTTCAGAAGGCCAAAC	CCTTGCTCAGTTACAGACTTCATGCA

Table S1: Bulk RNA-seq of hPSC-derived endothelial samples. Table of normalized raw RNA-seq counts filtered for |log2 fold change| >2.0 and adjusted p-value ≤0.05.
